# *Theobroma cacao* improves bone growth by modulating defective ciliogenesis in a mouse model of achondroplasia

**DOI:** 10.1038/s41413-021-00177-7

**Published:** 2022-01-25

**Authors:** Ludovic Martin, Nabil Kaci, Catherine Benoist-Lasselin, Marine Mondoloni, Suzanne Decaudaveine, Valentin Estibals, Maxence Cornille, Léa Loisay, Justine Flipo, Benoît Demuynck, Maria de la Luz Cádiz-Gurrea, Florent Barbault, Salvador Fernández-Arroyo, Laurent Schibler, Antonio Segura-Carretero, Emilie Dambroise, Laurence Legeai-Mallet

**Affiliations:** 1 Université de Paris, Imagine Institute, Laboratory of Molecular and Physiopathological Bases of Osteochondrodysplasia, INSERM UMR 1163, F‑75015, Paris, France; 2 Inovarion, Paris, France; 3 Department of Analytical Chemistry, University of Granada, Granada, Spain; 4 Research and Development of Functional Food Centre (CIDAF), Granada, Spain; 5 Université de Paris, ITODYS, CNRS, UMR 7086, 15 rue J-A de Baïf, Paris, France; 6 Biomedical Research Unit, Medicine and Surgery Department, Rovira i Virgili University, Tarragona, Spain; 7 ALLICE, Maison Nationale des Eleveurs, Paris, France

**Keywords:** Diseases, Bone

## Abstract

A gain-of-function mutation in the fibroblast growth factor receptor 3 gene (*FGFR3*) results in achondroplasia (ACH), the most frequent form of dwarfism. Constitutive activation of FGFR3 impairs bone formation and elongation and many signal transduction pathways. Identification of new and relevant compounds targeting the FGFR3 signaling pathway is of broad importance for the treatment of ACH, and natural plant compounds are prime drug candidate sources. Here, we found that the phenolic compound (-)-epicatechin, isolated from *Theobroma cacao*, effectively inhibited FGFR3’s downstream signaling pathways. Transcriptomic analysis in an *Fgfr3* mouse model showed that ciliary mRNA expression was modified and influenced significantly by the Indian hedgehog and PKA pathways. (-)-Epicatechin is able to rescue mRNA expression impairments that control both the structural organization of the primary cilium and ciliogenesis-related genes. In femurs isolated from a mouse model (*Fgfr3*^*Y367C/+*^) of ACH, we showed that (-)-epicatechin eliminated bone growth impairment during 6 days of ex vivo culture. In vivo, we confirmed that daily subcutaneous injections of (-)-epicatechin to *Fgfr3*^*Y367C/+*^ mice increased bone elongation and rescued the primary cilium defects observed in chondrocytes. This modification to the primary cilia promoted the typical columnar arrangement of flat proliferative chondrocytes and thus enhanced bone elongation. The results of the present proof-of-principle study support (-)-epicatechin as a potential drug for the treatment of ACH.

## Introduction

The most common form of dwarfism, achondroplasia (ACH), is caused by a gain-of-function mutation in the *FGFR3* gene encoding fibroblast growth factor receptor 3.^1^ FGFR3 gain-of-function mutations are also associated with hypochondroplasia, mild dwarfism, thanatophoric dysplasia (TD), and severe and lethal dwarfism.^2^

Defective FGFR3 signal transduction impairs intracellular downstream signaling pathways, including extracellular signal-regulated kinases 1 and 2 (ERK1/2), p38, phosphoinositide 3-kinase/protein kinase B (PI3K/AKT), phospholipase Cγ (PLCγ), and signal transducers and activators of transcription (STATs), thus affecting chondrocyte proliferation, differentiation, and bone elongation.^2^ FGFR3 plays a significant role in growth plate development, acting to inhibit both the rate of chondrocyte proliferation and differentiation and the interactions with the Indian hedgehog (Ihh) signaling pathway to control chondrocyte formation.^3^ Ihh signaling, which plays an essential role in chondrocyte differentiation, is fully dependent on primary cilia.^4^ FGF signaling also regulates the length of the primary cilia in many tissues.^5,6^ FGFR3 signaling interacts with Hedgehog and the serine/threonine kinase intestinal cell kinase (Ick), which is involved in ciliogenesis and participates in the control of ciliary length.^7,8^ For ACH, we demonstrated defective primary cilium elongation in mouse and human chondrocytes.^7–9^ The regular alignment of primary cilia is responsible for columnar-stacked chondrocytes in growth plate cartilage.^10^ The primary cilium is essential for the regulation of chondrocyte rotation, as demonstrated by the presence of defective primary cilium biosynthesis and/or function in many skeletal ciliopathies characterized by short ribs, short limbs and polydactyly.^11–13^ Interestingly, some clinical features of skeletal ciliopathies (short ribs and short limbs) are shared with achondroplasia.^9^

In recent years, a significant body of work has focused on treatments for ACH. Nonsurgical therapeutic strategies have been developed from insights gained from preclinical studies using *Ach* mouse models.^2,14^ There are many therapeutic approaches with various mechanisms, including: (i) targeting the FGF ligand (recifercept) and aptamer (APT-F2P/RBM 007), (ii) targeting FGFR (anti-FGFR3 antibody-B701/vofatamab), (iii) inhibiting tyrosine kinase activity (BGJ398/infigratinib), or (iv) using a C-type natriuretic peptide (CNP) (TransCon CNP, BMN111/vosoritide) analog^15^ to antagonize the mitogen-associated protein kinase (MAPK) pathway. The vosoritide approach is currently the furthest along in the clinical development pathway.^16,17^

Identification of new and relevant compounds targeting the FGFR3 signaling pathway is of broad importance for the treatment of FGFR3-related chondrodysplasia. Natural plant compounds are prime sources of drug candidates.^18^ Plant polyphenols such as *Theobroma cacao* contain flavon-3-ols and polyphenols that have long been considered to have relevant biological activities for the treatment of a number of diseases and are known to act in various ways on MAPK signaling pathways. For example, these compounds can: (i) reduce reactive oxygen species production and ERK1/2 and p38 phosphorylation in neurons,^19^ (ii) inhibit adipocyte differentiation through AMP-activated protein kinase (AMPK) and the ERK1/2 signaling pathways,^20^ and (iii) modulating antioxidant enzyme activities through ERK1/2 and regulating glucose production through AMPK modulation in liver cells.^21^

In this study, we identified (-)-epicatechin from a *Theobroma cacao* extract as a drug candidate. (-)-Epicatechin is able to (i) induce primary cilium elongation in chondrocytes and modify the differentially expressed genes in the growth plate cartilage of *Fgfr3*^*Y367C/+*^ mice, (ii) decrease FGFR3’s downstream signaling pathways in cell and organ cultures, and (iii) promote femur growth elongation in both femur explants and live *Fgfr3*^*Y367C/+*^ mice. Our results highlight this molecule’s specific action on the Ihh pathway related to primary cilia and suggest that (-)-epicatechin could be developed as a means to correct bone growth defects in patients with achondroplasia.

## Results

### *Theobroma cacao* extract restores abnormal activation of the FGFR3 pathway and primary cilium defects in mutant *Fgfr3* chondrocytes

To investigate the therapeutic efficacy of *Theobroma cacao* on the abnormal activation of the FGFR3 pathway, we fractionated a *Theobroma cacao* extract by combining solid-phase extraction with semipreparative HPLC^18^ and collected a total of 11 fractions (fractions 1 to 11) (Supplementary Fig. 1). The activities of the fractions were determined according to two relevant criteria considered a hallmark of FGFR3-related disorders, namely, (i) the ability to inhibit the MAPK pathway, a downstream effector of the activated FGFR3 pathway,^22^ and (ii) the ability to promote primary cilia elongation.^7,9^

**Fig. 1:**
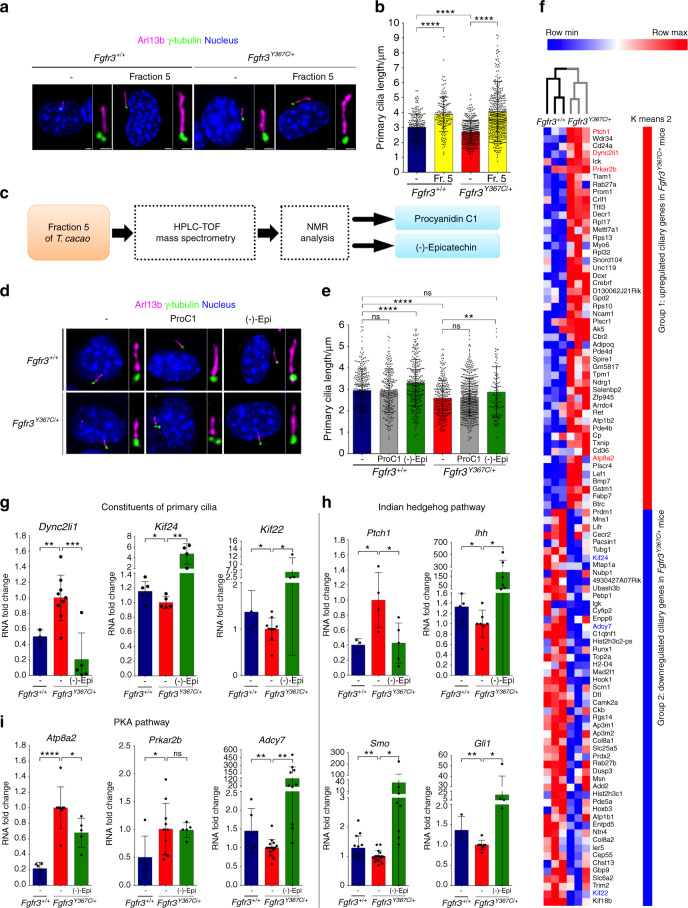
(-)-Epicatechin from *Theobroma cacao* fraction 5 modifies both ciliary length and ciliary gene expression. **a** Representative confocal microscopy image of the primary cilia of E16.5 chondrocytes immunolabeled with Arl13b (magenta) for axoneme, γ-tubulin (green) for the basal body, and DAPI (blue) for cell nuclei. **b** Graphical representation of the length of the primary cilium in E16.5 *Fgfr3*^*+/+*^ (*n* = 219 and *n* = 162 for exposed cells) and *Fgfr3*^*Y367C/+*^ murine chondrocytes exposed (*n* = 338) or not (*n* = 443) to fraction 5 of *Theobroma cacao*. **c** Sequential representation of the identification of two major compounds (procyanidin C1 and (-)-epicatechin) in *Theobroma cacao* fraction 5. **d** Representative confocal microscopy image of the E16.5 primary cilium immunolabeled with Arl13b (magenta), γ-tubulin (green), and DAPI (blue). **e** Graphical representation of the length of the primary cilium in E16.5 *Fgfr3*^*+/+*^ not exposed (2.95 ± 0.06; *n* = 300), exposed to procyanidin C1 (2.94 ± 1.02; *n* = 232) or exposed to (-)-epicatechin (3.30 ± 1.10; *n* = 278) and *Fgfr3*^*Y367C/+*^ murine chondrocytes not exposed (2.58 ± 0.82; *n* = 272), exposed to procyanidin C1 (2.62 ± 0.87; *n* = 461) or exposed to (-)-epicatechin (2.87 ± 1.18; *n* = 113). **f** Heatmap from a comparative transcriptome analysis of the upregulated (red) and downregulated (blue) genes in growth plate cartilage from 1-week-old *Fgfr3*^*+/+*^ and *Fgfr3*^*Y367C/+*^ mice. **g** RNA expression fold change for *Dync2li1*, *Kif24*, and *Kif22* mRNA. **h** Fold change in *Ptch1*, *Ihh*, *Smo*, and *Gli1* mRNA expression. **i** RNA expression fold change for *Atp8a2*, *Prkar2*, and *Adcy7* mRNA. Data are presented as the mean ± SEM. ns not significant; *P < 0.05; **P < 0.01; ***P < 0.001; ****P < 0.000 1 by two-tailed, unpaired *t* test

We found by western blot that *Theobroma cacao* extract fraction 5 significantly lowered ERK1/2 phosphorylation in human ACH and TD chondrocytes (Supplementary Fig. 2a, b). Moreover, we found by immunolabeling chondrocytes isolated from *Fgfr3*^*Y367C/+*^ mice exhibiting a dwarf phenotype^23^ that fraction 5 treatment increased the initial primary cilium length defects in *Fgfr3*^*Y367C/+*^ chondrocytes (4.06 ± 2.15 µm) compared to nontreated *Fgfr3*^*Y367C/+*^ chondrocytes (2.71 ± 0.75 µm) (Fig. 1a, b).

### Two compounds, procyanidin C1, and (-)-epicatechin, are present in fraction 5

To identify the putative dual-effect compound, the compounds comprising fraction 5 from *Theobroma cacao* were separated and purified by an HPLC system coupled to a time-of-flight (TOF) mass spectrometer and analyzed by NMR (Fig. 1c). NMR analyses revealed the presence of two different compounds: procyanidin C1 (10%–20%) and (-)-epicatechin (80%–90%) (Fig. 1c and Supplementary Fig. 3a–e). To evaluate the abilities of procyanidin C1 and (-)-epicatechin to act on primary cilium elongation, we measured the lengths of the primary cilia in in vitro *Fgfr3*^*Y367C/+*^ and *Fgfr3*^*+/+*^ cultured mouse chondrocytes (Fig. 1d). Treatment with procyanidin C1 (at a range of concentrations, not shown) failed to increase primary cilium length in both *Fgfr3*^*Y367C/+*^ (2.62 ± 0.87 µm) and *Fgfr3*^*+/+*^ chondrocytes (2.94 ± 1.02 µm) compared to nontreated *Fgfr3*^*Y367C/+*^ (2.58 ± 0.82 µm) and nontreated *Fgfr3*^*+/+*^ chondrocytes (2.95 ± 1.00 µm), respectively (Fig. 1d, e). Interestingly, treatment with (-)-epicatechin rescued the primary cilium length defect in *Fgfr3*^*Y367C/+*^ chondrocytes; the mean length (2.87 ± 1.18 µm) was similar to that measured in nontreated *Fgfr3*^*+/+*^ chondrocytes (2.95 ± 1.00 µm) (Fig. 1d, e). Thus, we concluded that among the two compounds ((-)-epicatechin and procyanidin C1) contained in *Theobroma cacao* fraction 5, only purified (-)-epicatechin showed beneficial effects on primary cilium length.

### Transcriptomic identification of the primary cilium genes in cartilage

The mechanism by which FGF signaling controls cilia in growth plate cartilage is not well understood. Here, to define relevant genes controlling ciliogenesis in FGFR3-related dwarfism, we performed a comparative, longitudinal, transcriptomic analysis of RNA isolated from cartilage growth plates of *Fgfr3*^*Y367C/+*^ mice *versus* controls. After the identification of 3336 modulated probe sets, 763 differentially expressed probe sets (612 genes) were found to be significantly modulated (absolute fold change >1.5 and an adjusted *P* value ≤5% relative to the overall changes in expression) and were fed into a functional analysis (Gene Expression Omnibus, database ID: GSE145821). Of these, 102 genes were shown to be related to primary cilia (Gene Expression Omnibus database ID: GSE145821, Gene Ontology annotation and/or registration in the Cildb primary cilia gene database).^24^ As shown in Fig. 1f, hierarchical clustering of the expression levels led to the identification of two groups of genes: those upregulated (group 1) and those downregulated (group 2). In agreement with previous data, we found that Ick was upregulated,^8^ and we noted the relevant upregulation of primary cilia genes such as Ptch1 (protein patched homolog 1), Dync2li1 (coding for dynein 2 light intermediate chain 1, a regulator of primary cilium length), Prkar2b (protein kinase camp-dependent type II regulatory subunit β), and Atp8a2 (ATPase phospholipid transporting 8A2). Interestingly, we observed the downregulation of Kif24 (kinesin family member 24), Adcy7 (adenylated cyclase 7) and Kif22 (kinesin family member 22). Among the selected upregulated or downregulated genes, we were able to distinguish three families of genes, those associated with (i) cilium components, (ii) the Hh (Hedgehog) pathway, and (iii) the protein kinase A (PKA) pathway (Fig. 1f). In an attempt to further understand the mechanisms underlying (-)-epicatechin’s effects on the primary cilium, using an in vitro test, we monitored gene expression in *Fgfr3*^*Y367C/+*^ chondrocytes treated or not with (-)-epicatechin (Fig. 1g–i). We first evaluated the genes *Dync2li1*, *Kif24*, and *Kif22* involved in cilia formation and skeletal ciliopathies (Fig. 1g).^25–27^ *Dync2li1* is highly expressed in *Fgfr3*^*Y367C/+*^ chondrocytes,^27^ and this expression was significantly reduced (4.8-fold) by (-)-epicatechin treatment. The lower expression levels of *Kif24* and *Kif22* observed in *Fgfr3*^*Y367C/+*^ growth plates (Fig. 1f) were increased by 4.7- and 6.1-fold, respectively, after (-)-epicatechin treatment to *Fgfr3*^*Y367C/+*^ chondrocytes (Fig. 1g). Second, we investigated the Hh pathway commonly dysregulated in ciliopathies^28,29^ (Fig. 1h). It is well established that primary cilia play a central role in Ihh signal transduction. Ihh binds to the Ptch1 receptor to release Smo (*Smoothened)*, a signal transducer from Patched-dependent suppression. Smo stabilizes Gli (*Gli family zinc finger)* family members and promotes the nuclear accumulation of Gli. The elevated mRNA expression of *Patch1* in *Fgfr3*^*Y367C/+*^ chondrocytes (Fig. 1f) was notably reduced by 2.3-fold in (-)-epicatechin-treated chondrocytes (Fig. 1i). The downregulated expression of *Ihh* and successive downstream effectors of *Ptch1* (*Smo* and *Gli1*) in *Fgfr3*^*Y367C/+*^ chondrocytes compared to controls were strongly increased by (-)-epicatechin treatment (by 191.7-, 62.6-, and 16.5-fold, respectively) (Fig. 1h). We also investigated the PKA pathway as a regulator of Hh signaling.^30^ Interestingly, *Atp8a2* overexpression in mutant chondrocytes was reduced 1.5-fold by (-)-epicatechin treatment (Fig. 1i). In contrast, overexpression of *Prkar2b* (another member of the PKA-dependent pathway) in *Fgfr3*^*Y367C/+*^ chondrocytes was not modified by treatment (Fig. 1i). Furthermore, we observed that *Adcy7* expression increased 92.7-fold after (-)-epicatechin treatment (Fig. 1i). Our results suggest that in *Fgfr3*-mutated chondrocytes, (-)-epicatechin is able to rescue impairments in the expression of (i) key genes that control both the structural organization of the primary cilium and (ii) ciliogenesis-related genes that act on the Ihh and PKA signaling pathways involved in the regulation of cartilage homeostasis in vitro.

### (-)-Epicatechin counteracts the abnormal activation of the FGFR3 signaling pathway

**Fig. 2:**
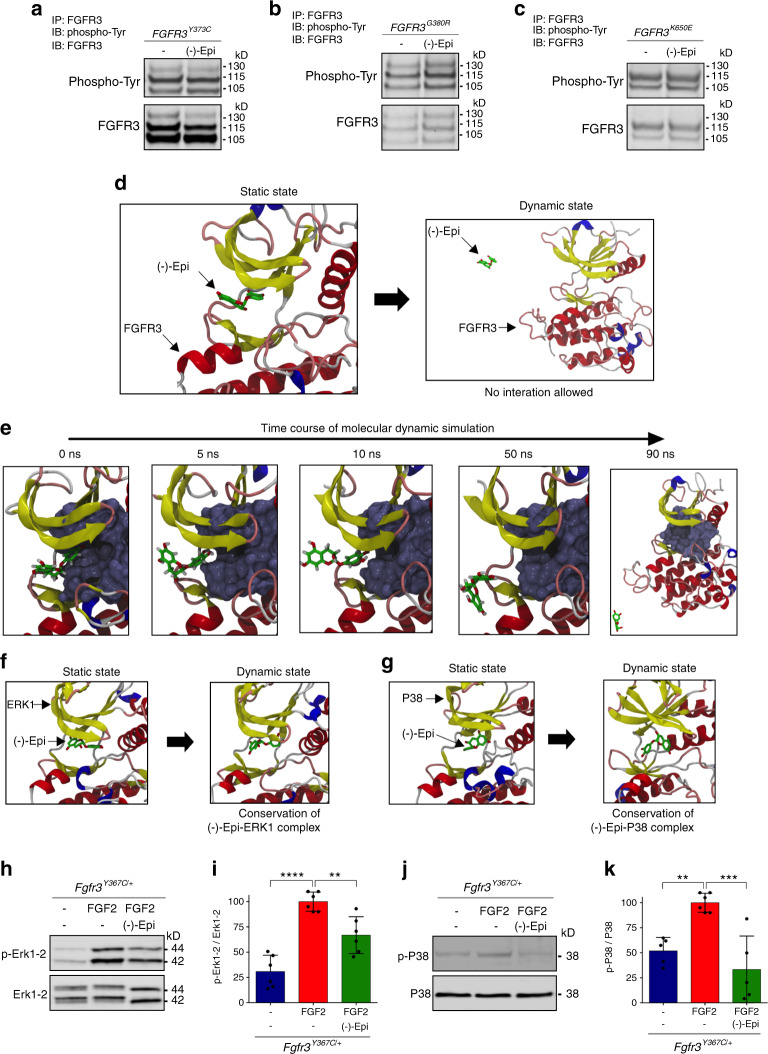
(-)-Epicatechin acts downstream of FGFR3 on cartilage homeostasis. **a**, **b**, **c** Representative western blots of tyrosine-phosphorylated FGFR3 (p-Tyr) expression from FGFR3 immunoprecipitated from HEK293 cells transfected with *FGFR3*^*Y373C*^ (**a**), *FGFR3*^*G380R*^ (**b**), and *FGFR3*^*K650E*^ (**c**) exposed or not to (-)-epicatechin. **d**–**g** Computational results of (-)-epicatechin binding to FGFR3. In all these images, the protein is displayed as ribbons (yellow: β-sheet, red: α-helix, pink: turn and white: random coil), and the ATP active site is presented as a molecular surface in ice blue. (-)-Epicatechin is displayed in CPK colors with carbon in green. Structures on the top (**d**, top) correspond to the FGFR3-(-)-epicatechin complex after molecular docking. The hydrogen bonds between interacting residues (presented with gray carbon atoms) are shown with dashed lines. The structure on the bottom (**d**, bottom) was obtained after molecular dynamics and hyperdynamics simulations. **e** Detailed illustrations of the dissociation of (-)-epicatechin from FGFR3 kinase. From left to right, snapshots were extracted after 0, 5, 10, and 50 ns of classical molecular dynamics and after 40 ns of hyperdynamics (see Supplemental Movie 1). **f**, **g** Representative structures of (-)-epicatechin complexed with the kinases ERK1 (**f**), ERK2 (Supplementary Fig. 4) and p38 (**g**). The complexes obtained after molecular docking (i.e., static states) are shown on the left, and those obtained after the molecular dynamics and hyperdynamics simulations (i.e., dynamic states) are shown on the right. The ATP-binding sites are presented as molecular surfaces in ice blue. **h** Representative western blots of the expression levels of p-ERK1/2 and ERK1/2 in primary chondrocytes isolated from the femoral cartilage of *Fgfr3*^*Y367C/+*^mice. **i** Changes in the ratio of p-ERK1/2 to ERK1/2 (*n* = 5). **j** Representative western blots of the expression levels of p-p38 and p38 in primary chondrocytes isolated from the femoral cartilage of *Fgfr3*^*Y367C/+*^mice. **k** Changes in the ratio of p-p38 to p38 (*n* = 5). Data are presented as the mean ± SD. ns, not significant; *P < 0.05; **P < 0.01; ***P < 0.001; ****P < 0.000 1 by two-tailed, unpaired *t* test. The gray density analysis for the western blot data was performed by using ImageJ software

(-)-Epicatechin has shown beneficial effects on primary cilia formation in *Fgfr3*^*Y367C/+*^ chondrocytes. Therefore, we transiently transfected human embryonic kidney 293 (HEK293) cells with FGFR3 constructs bearing a gain-of-function mutation in the protein’s extracellular domain (TD/Y373C; Fig. 2a), transmembrane domain (ACH/G380R; Fig. 2b), or intracellular domain (TD/K650E; Fig. 2c) to determine whether (-)-epicatechin acts directly on FGFR3 phosphorylation. FGFR3 phosphorylation in the mutants was not modified by (-)-epicatechin treatment (Fig. 2a–c). We next performed in silico analysis to define the ability of (-)-epicatechin to bind to the FGFR3 kinase binding pocket. Visualization of the structural model clearly showed that (-)-epicatechin does not fully insert into FGFR3’s ATP-binding pocket. To determine the ability of (-)-epicatechin to bind to the kinase target, a molecular dynamics simulation (duration: 50 ns) was undertaken for each (-)-epicatechin complex. The results of the hyperdynamics calculations (static state to dynamic state, Fig. 2d) revealed that (-)-epicatechin was unable to form a favorable complex with human FGFR3 and was gradually pushed away from the FGFR3 binding pocket (Fig. 2d, bottom panel and Supplementary Table 1). During the first 10 ns (Fig. 2e, left panel), the hydrogen bonds between (-)-epicatechin and Y557, E556, and A558 broke slowly, exposing more (-)-epicatechin to the solvent (water). At the end of the conventional 50 ns molecular dynamics step, (-)-epicatechin was still at the surface of the FGFR3 protein and too far from the kinase’s active site (Fig. 2e, last right panel) to act as an inhibitor (for detailed animation, please see Supplementary Movie 1). In light of these results, we performed similar experiments with procyanidin C1, and we observed that this large molecule did not interact with FGFR3 (Supplementary Fig. 4 and Supplementary Table 2).

The defective bone elongation observed in FGFR3-related disorders is due, at least in part, to dysregulation of chondrocyte differentiation. The successive differentiation steps are regulated by the ERK–MAPK pathway.^31^ Here, we evaluated the propensity of (-)-epicatechin to interact with FGFR3 downstream signaling pathways (several distinct MAPKs, including ERK1, ERK2, and p38 kinases). We found that (-)-epicatechin remained bound to ERK1, ERK2, and p38 with only small structural variations from the starting dynamic state (static state) (Fig. 2f, g, Supplementary Fig. 5, and Supplementary Table 1). To validate the interactions of (-)-epicatechin with FGFR3’s downstream signaling pathways in cartilage cells, we quantified the levels of ERK1/2 and p38 phosphorylation in primary chondrocytes isolated from *Fgfr3*^*Y367C/+*^ mice^23^ treated with (-)-epicatechin. Compared with untreated *Fgfr3*^*Y367C/+*^ chondrocytes, the levels of ERK1/2 phosphorylation (Fig. 2h, i) and p38 phosphorylation (Fig. 2j, k) were significantly lower in mutant chondrocytes treated with (-)-epicatechin. To definitively eliminate procyanidin C1 as a drug candidate, in silico analyses demonstrated that procyanidin C1 failed to interact with ERK1, ERK2, and p38 (Supplementary Fig. 6 and Supplementary Table 2) and did not reduce the level of ERK1/2 phosphorylation in either primary *Fgfr3*^*Y367C/+*^ murine or human *FGFR3-Y373C* chondrocytes (data not shown). These findings indicate that (-)-epicatechin might be a promising compound for repressing the constitutive activation of FGFR3’s downstream signaling pathways in cartilage cells.

### (-)-Epicatechin enhances bone growth and regulates ciliogenesis and growth plate organization

**Fig. 3:**
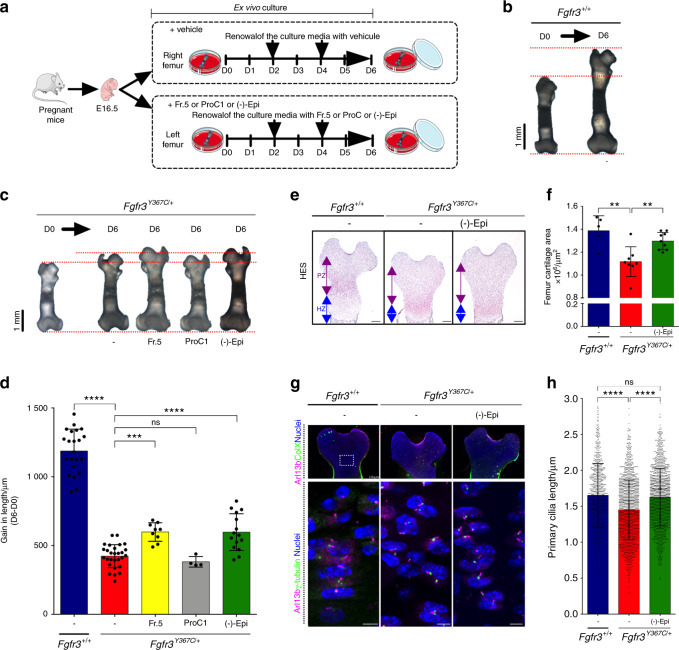
(-)-Epicatechin increases ex vivo *Fgfr3*^*Y367C/+*^ femur growth through primary cilia elongation. **a** Sequential representation of the ex vivo culture of femurs isolated from mouse embryos (E16.5). **b** Representative images of E16.5 *Fgfr3*^*+/+*^ femurs in ex vivo culture for 0 (D0) and 6 (D6) days. **c** Representative images of E16.5 *Fgfr3*^*Y367C/+*^ femurs in ex vivo culture for 0 (D0) and 6 (D6) days exposed (or not) to *Theobroma cacao* fraction 5, procyanidin C1 or (-)-epicatechin. **d** Graphical representation of the gain in length of the ex vivo E16.5 *Fgfr3*^*+/+*^ and *Fgfr3*^*Y367C/+*^ femurs after exposure (or not) to *Theobroma cacao* fraction 5, procyanidin C1 or (-)-epicatechin (*n* = 15). **e** Representative images of HES-stained immunostained embryonic distal femurs. Scale bars: 200 μm. PZ proliferative zone, HZ hypertrophic zone. **f** Graphical representation of the femur growth plate area in E16.5 *Fgfr3*^*+/+*^ (*n* = 5) and *Fgfr3*^*Y367C/+*^ femurs in the presence (*n* = 8) or absence (*n* = 7) of (-)-epicatechin. **g** Representative confocal image of the primary cilium immunolabeled with Arl13b (magenta), type X collagen or γ-tubulin (green), and DAPI (blue) localized in the control and *Fgfr3*^*Y367C/+*^ femurs at Day 6. **h** Graphical representation of the primary cilium length in the chondrocytes from femur cultures at Day 6 from *Fgfr3*^*+/+*^ (*n* = 917) and *Fgfr3*^*Y367C/+*^ femurs exposed (*n* = 1 667) or not (*n* = 1 575) to (-)-epicatechin. Data are presented as the mean ± SD. ns not significant; *P < 0.05; **P < 0.01; ***P < 0.001; ****P < 0.000 1 by two-tailed, unpaired *t* test

To assess the relevance of using polyphenol compounds to treat cartilage tissue, we compared the growth of femurs isolated from dwarf *Fgfr3*^*Y367C/+*^ embryos (E16.5)^23^ in ex vivo cultures for 6 days in the presence of fraction 5 from *Theobroma cacao* (positive control), procyanidin C1 (used as a negative control), (-)-epicatechin and vehicle (Fig. 3a). The mean growth was 1 188 ± 159 µm for the *Fgfr3*^*+/+*^ (Fig. 3b) and 423 ± 84 µm for *Fgfr3*^*Y367C/+*^ explant femurs at Day 6 (Fig. 3c). The growth of the (-)-epicatechin-treated *Fgfr3*^*Y367C/+*^ femurs was quite similar to that of fraction 5-treated *Fgfr3*^*Y367C/+*^ femurs (598 ± 134 µm), whereas procyanidin C1-treated *Fgfr3*^*Y367C/+*^ femurs (382 ± 38 µm) remained unchanged compared to *Fgfr3*^*Y367C/+*^ untreated femurs (Fig. 3c, d). (-)-Epicatechin treatment for 6 days improved the growth of the mutant *Fgfr3*^*Y367C/+*^ femurs by 1.41-fold. At the end of treatment, examination of the cartilage’s general layout and cell distribution revealed that the area of *Fgfr3*^*Y367C/+*^ femoral cartilage in the treated distal and proximal femurs was larger (1.30 × 10^6^ ± 0.08 × 10^6^ µm^2^) than that in the untreated femurs (1.12 × 10^6^ ± 0.13 × 10^6^ µm^2^) (Fig. 3e, f). Using frozen sections of ex vivo control and mutant femur growth plates, we examined the primary cilia length in the type X collagen-negative (proliferative) zone (Fig. 3g, h), and confirmed that the primary cilium length was shorter in the *Fgfr3*^*Y367C/+*^ proliferative zone (1.46 ± 0.41 µm) than in the control *Fgfr3*^*+/+*^ (1.65 ± 0.44 µm) (Fig. 3g, h). Treatment with (-)-epicatechin corrected the primary cilium length of *Fgfr3*^*Y367C/+*^ chondrocytes (1.63 ± 0.40 µm) to a length similar to that of the *Fgfr3*^*+/+*^ control chondrocytes (Fig. 3g, h). Measuring the x–y major axis of the cell’s longitudinal position and the angle ϕ with respect to the cell’s x–y axis, we observed that the angle ϕ was lower (27.1 ± 19.8°) in the *Fgfr3*^*Y367C/+*^ growth plate, suggesting that the primary cilium was unable to erect in the proliferative growth plate chondrocytes (Supplementary Fig. 7). (-)-Epicatechin restored the angle ϕ (39.7 ± 24.2°) to values close to those seen in *Fgfr3*^*+/+*^ experiments (41.6 ± 27.1°) (Supplementary Fig. 7).

### (-)-Epicatechin modulates cell proliferation and differentiation in ACH growth plate cartilage

**Fig. 4:**
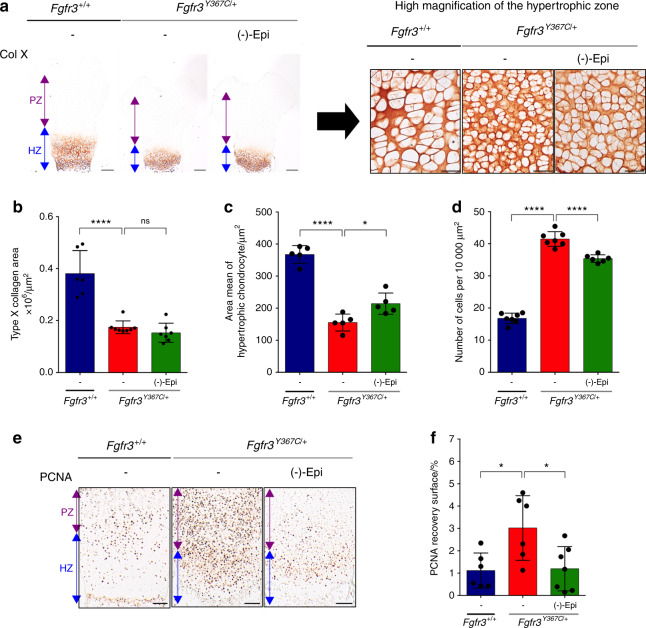
(-)-Epicatechin modulates hypertrophic and proliferative chondrocytes in ex vivo *Fgfr3*^*Y367C/+*^ femurs. **a** Representative images of type X collagen immunostaining of *Fgfr3*^*+/+*^ and *Fgfr3*^*Y367C/+*^ embryonic distal femurs (scale bars: 200 μm). High-magnification inset of a representative image of type X collagen immunostaining in the hypertrophic distal zone of *Fgfr3*^*+/+*^ and *Fgfr3*^*Y367C/+*^ distal femurs (scale bars: 50 μm). **b** Graphical representation of the type X collagen area immunostaining in E16.5 *Fgfr3*^*+/+*^ (*n* = 5) and *Fgfr3*^*Y367C/+*^ distal femurs in the presence (*n* = 8) or absence (*n* = 7) of (-)-epicatechin. **c** Graphical representation of the area of each hypertrophic chondrocyte of *Fgfr3*^*+/+*^ (*n* = 5) and *Fgfr3*^*Y367C/+*^ distal femurs in the presence (*n* = 5) or absence (*n* = 5) of (-)-epicatechin. **d** Graphical representation of the number of hypertrophic chondrocytes per 10 000 µm^2^ of surface area from *Fgfr3*^*+/+*^ (*n* = 80) and *Fgfr3*^*Y367C/+*^ distal femurs in the presence (*n* = 80) or absence (*n* = 5) of (-)-epicatechin. **e** PCNA immunostaining of *Fgfr3*^*+/+*^ and *Fgfr3*^*Y367C/+*^ distal femurs. Scale bars: 100 μm. **f** Graphical representation of the PCNA recovery surface of E16.5 *Fgfr3*^*+/+*^ (*n* = 5) and *Fgfr3*^*Y367C/+*^ distal femurs in the presence (*n* = 8) or absence (*n* = 7) of (-)-epicatechin. PZ proliferative zone (purple arrow), HZ hypertrophic zone (blue arrow). Data are presented as the mean ± SD. ns not significant; *P < 0.05; **P < 0.01; ***P < 0.001; ****P < 0.000 1 by two-tailed, unpaired *t* test

To determine which cartilage marker proteins are targeted by (-)-epicatechin to promote femur elongation, we examined (-)-epicatechin’s putative actions on chondrocyte differentiation (as characterized by changes in cell morphology and collagen expression). Type X collagen expression, a marker of late-stage hypertrophic chondrocyte differentiation, was decreased in *Fgfr3*^*Y367C/+*^ femurs.^23^ By quantitative immunolabeling of collagen type X (Fig. 4a, b), we did not see a significant change in the overall size of the hypertrophic zone after (-)-epicatechin treatment (1.5 × 10^5^ ± 0.4 × 10^5^ µm^2^) (Fig. 4b). As shown in the high-magnification inset image of the type X collagen-positive zone (Fig. 4a, right panel), we found that the hypertrophic cells were 38.1% larger in the (-)-epicatechin-treated ex vivo *Fgfr3*^*Y367C/+*^ femurs (214 ± 33 µm^2^) than in the nontreated femurs (155 ± 26 µm^2^) (Fig. 4a, c). We also noted that the number of cells per surface was doubled in the nontreated *Fgfr3*^*Y367C/+*^ growth plate cartilage (41.5 ± 2.3 chondrocytes per 10 000 µm^2^) compared to the *Fgfr3*^*+/+*^ growth plate cartilage (16.8 ± 1.6 chondrocytes per 10 000 µm^2^). After 6 days of ex vivo culture, the number of cells per surface decreased significantly in (-)-epicatechin-treated *Fgfr3*^*Y367C/+*^ femurs (35.4 ± 1.2 chondrocytes per 10 000 µm^2^; Fig. 4a, d), suggesting that (-)-epicatechin treatment can, to some extent, partially rescue the defective final differentiation of the collagen type X-positive cells in the *Fgfr3*^*Y367C/+*^ growth plate.

We then hypothesized that (-)-epicatechin treatment mainly stimulated proliferative chondrocytes in growth plate cartilage. To investigate chondrocyte proliferation, we immunolabeled the proliferating cell nuclear antigen (PCNA) protein in ex vivo femur cultures (Fig. 4e). Elevated expression of PCNA in mutant growth plates was reported, as *Fgfr3* mutation induces cell cycle exit and promotes abnormal chondrocyte differentiation.^22^ As previously observed,^22^ we noted that PCNA was overexpressed in the *Fgfr3*^*Y367C/+*^ cartilage growth plate (3.02% ± 1.45% of the PCNA recovery surface) relative to the control (1.12% ± 0.79%) (Fig. 4e, f). Here, we found that PCNA expression levels were lower in (-)-epicatechin-treated *Fgfr3*^*Y367C/+*^ femurs (1.20% ± 0.99% vs. 3.02% ± 1.45%) (Fig. 4f). Treatment with (-)-epicatechin for 6 days countered the abnormally high expression of PCNA, indicating that (-)-epicatechin normalizes chondrocyte proliferation blockade and promotes chondrocyte maturation in *Fgfr3*^*Y367C/+*^ femurs.

We also hypothesized that (-)-epicatechin treatment could stimulate chondrocyte differentiation in growth plate cartilage. Chondrocyte differentiation is driven by many genes required to maintain healthy cartilage. The MAPK pathway (including p38 and ERK1/2) affects Sox9 transcription, chondrocyte differentiation, and cartilage matrix synthesis.^32^ Sox9, along with several other transcription factors, could also be regulated by p38, and elevated levels of phosphorylated p38 promote hypertrophic chondrocyte differentiation.^33^ Here, we observed that abnormal activation of FGFR3 signaling promotes abnormally high Sox9 expression (as previously described^34,35^) and p38 and ERK1/2 phosphorylation levels in the growth plates of the cartilage of the femur explants. Interestingly, treatment with (-)-epicatechin was associated with lower levels of Sox9, phosphorylated p38 and ERK1/2 in the *Fgfr3*^*Y367C/+*^ femur growth plate (Supplementary Fig. 8a–c). Overall, these proof-of-principle experiments showed that (-)-epicatechin modulates the activation of FGFR3’s major downstream signaling pathways.

Altogether, these data show that (-)-epicatechin has a beneficial effect on the proliferation/differentiation balance in *Fgfr3*^*Y367C/+*^ chondrocytes.

### (-)-Epicatechin modulates the architecture of bone and primary cilia in the *Fgfr3* mouse model of ACH

**Fig. 5:**
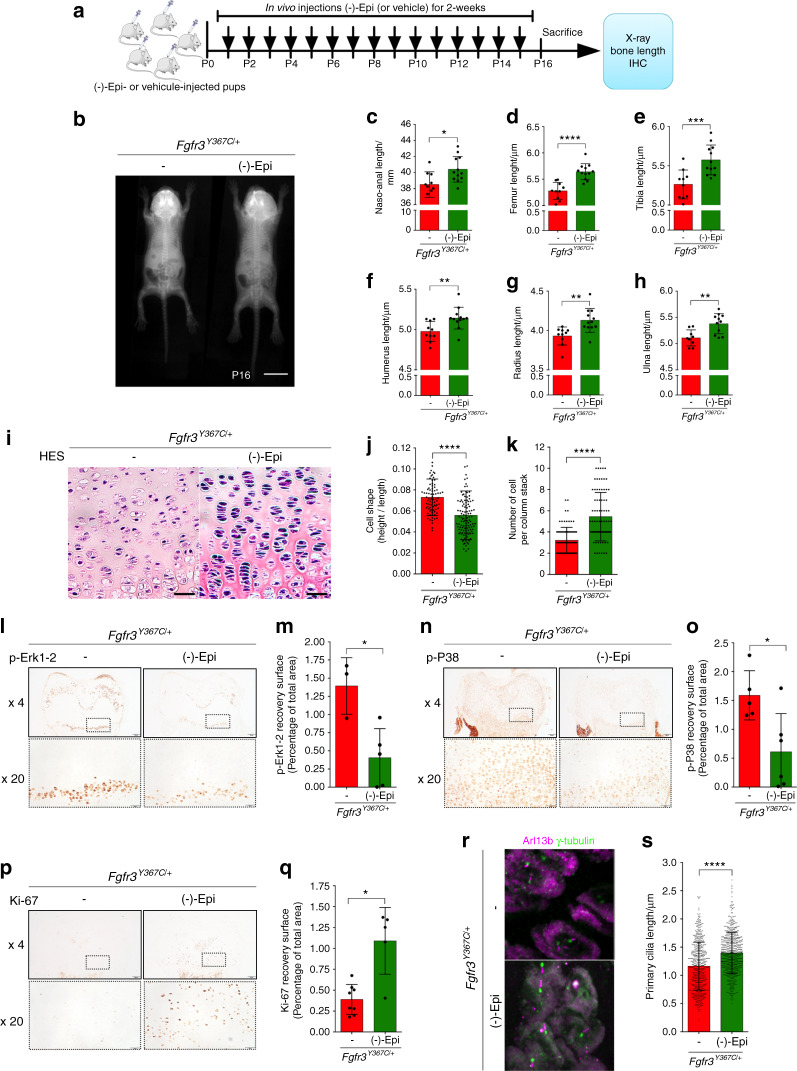
In vivo-injected (-)-epicatechin increases bone growth, modifies chondrocyte proliferation, and differentiation and promotes primary cilium elongation. **a** Sequential representation of the in vivo protocol. **b** Radiographs of *Fgfr3*^*Y367C/+*^ mice treated with vehicle or (-)-epicatechin for 16 days and graphical representations of the naso-anal lengths (**c**), femur lengths (**d**), tibia lengths (**e**), humerus lengths (**f**), radius lengths (**g**), and ulna lengths (**h**). **i** Representative HES staining images of the distal femurs. Scale bars: 50 μm. **j** Graphical representation of the cell height/cell length ratio in the growth plate proliferative zone in *Fgfr3*^*+/+*^ (*n* = 80) and *Fgfr3*^*Y367C/+*^ mice treated (*n* = 80) or not (*n* = 120) with (-)-epicatechin for 16 days. **k** Number of cells per column stack in the growth plate proliferative zone in *Fgfr3*^*Y367C/+*^ mice treated (*n* = 6) or not (*n* = 6) with (-)-epicatechin for 16 days. **l** Representative image of p- ERK1/2 in *Fgfr3*^*Y367C/+*^ distal femurs in the presence (*n* = 5) or absence (*n* = 5) of (-)-epicatechin. Scale bars: 200 μm. **m** Graphical representation of the surface of the p-ERK1/2-positive zone in *Fgfr3*^*Y367C/+*^ distal femurs in the presence (*n* = 5) or absence (*n* = 5) of (-)-epicatechin. **n** Representative image of p-p38 in *Fgfr3*^*Y367C/+*^ distal femurs in the presence (*n* = 5) or absence (*n* = 5) of (-)-epicatechin. Scale bars: 200 μm. **o** Graphical representation of the surface of the p-p38-positive zone. **p** Representative image of Ki-67 in *Fgfr3*^*Y367C/+*^ distal femurs in the presence (*n* = 5) or absence (*n* = 5) of (-)-epicatechin. Scale bars: 200 μm. **q** Graphical representation of the surface of the Ki-67-positive zone in *Fgfr3*^*Y367C/+*^ distal femurs treated or not with (-)-epicatechin. **r** Representative confocal microscopy images of primary chondrocytes from in vivo *Fgfr3*^*Y367C/+*^ femoral growth plates exposed or not to (-)-epicatechin. **s** Graphical representation of the length of the primary cilium in chondrocytes after in vivo treatment (see also Supplementary Fig. 8 for enlarged images). PZ proliferative zone (purple arrow), HZ hypertrophic zone (blue arrow). Data are presented as the mean ± SD. ns not significant; *P < 0.05; **P < 0.01; ***P < 0.001; ****P < 0.000 1 by two-tailed, unpaired *t* test

To confirm (-)-epicatechin’s in vivo effects on bone growth, we next sought to determine whether (-)-epicatechin could regulate bone growth in the *Fgfr3*^*Y367C/+*^ murine model of ACH.^23^ *Fgfr3*^*Y367C/+*^ mice were 1-day-old upon treatment initiation. Subcutaneous injections of (-)-epicatechin (0.1 mg·kg^−1^ per day) or vehicle were administered daily to *Fgfr3*^*Y367C/+*^ mice for 2 weeks (Fig. 5a), and we then acquired X-ray images of the animals (Fig. 5b). The mean naso-anal length was 4.9% greater in treated animals than in controls (38.51 ± 1.58 µm and 40.40 ± 1.59 µm for nontreated and (-)-epicatechin-treated *Fgfr3*^*Y367C/+*^ mice, respectively; Fig. 5c and Table 1). With regard to long bone growth, the femurs, and tibias increased their lengths by 7.02% (*P* < 0.000 1) and 5.89% (*P* < 0.001) in (-)-epicatechin-treated *Fgfr3*^*Y367C/+*^ mice compared to controls, respectively (Fig. 5d, e and Table 1). Similarly, the lengths of the humeri, radii, and ulnas in the treated *Fgfr3*^*Y367C/+*^ mice were 3.21% (*P* < 0.01), 5.09% (*P* < 0.01), and 5.28% (*P* < 0.01) longer, respectively, than those in the untreated controls (Fig. 5f–h and Table 1).

**Table 1:** (-)-Epicatechin increases bone growth in vivo

	Naso-anal length/mm	Femur length/mm	Tibia length/mm	Humerus length/mm	Radius length/mm	Ulna length/mm
Vehicle	38.51	5.27	5.26	4.98	3.93	5.11
(-)-Epicatechin	40.40	5.64	5.57	5.14	4.13	5.38
Δ Increase	+1.89 mm	+0.37 mm	+0.31 mm	+0.16 mm	+0.20 mm	+0.27 mm
Percentage of increase	+4.91%	+7.02%	+5.89%	+3.21%	+5.09%	+5.28%
*P* value	0.011	9.10^−10^	2.10^−6^	8.10^−4^	7.10^−5^	9.10^−4^
Significance	*	****	****	****	****	****

The percent growth gains of the naso-anal, femur, tibia, humerus, ulna, and radius lengths with detailed values for (-)-epicatechin-injected *Fgfr3*^*Y367C/+*^ animals. Data are presented as the mean

*ns* Not significant

*P < 0.05; **P < 0.01; ***P < 0.001; ****P < 0.000 1 by two-tailed, unpaired *t* test

The chondrocytes from the *Fgfr3*^*Y367C/+*^ proliferative zone were small, round and not structured into columns. (-)-Epicatechin’s in vivo mode of action was characterized by modification of the structural organization of the *Fgfr3*^*Y367C/+*^ growth plate cartilage; thus, the chondrocytes were more aligned, flattened, and oriented in stacks (Fig. 5i). The *Fgfr3*^*Y367C/+*^ chondrocytes were significantly flatter after 2 weeks of treatment with (-)-epicatechin (ratio of 7.3 × 10^2^ ± 0.2 × 10^2^ compared to the nontreated *Fgfr3*^*Y367C/+*^ mice (5.6 × 10^2^ ± 0.2 × 10^2^); Fig. 5j), and the number of cells per stack in the proliferative zone also increased after (-)-epicatechin treatment (5.4 ± 2.3 cells per stack compared to nontreated *Fgfr3*^*Y367C/+*^ mice (3.2 ± 1.2 cells per stack); Fig. 5k). In vivo administration of (-)-epicatechin decreased the expression of FGFR3 downstream effectors (ERK1/2, p38) (Fig. 5i–o) and promoted proliferation (Ki-67) (Fig. 5p, q).

In vivo (-)-epicatechin treatment also has a beneficial effect on primary cilia. We found that 2 weeks of (-)-epicatechin treatment was associated with markedly longer primary cilia in proliferating *Fgfr3*^*Y367C/+*^ chondrocytes (1.40 ± 0.37 µm) (Fig. 5r, s) and an increase in the ϕ angle value was observed (39.7 ± 24.2°) (Supplementary Fig. 9).

These findings confirmed that in vivo, systemically administered (-)-epicatechin penetrated the growth plate cartilage, strongly downregulated the expression of key regulators of chondrocyte proliferation and differentiation and modified the length of the primary cilia of chondrocytes. These results demonstrate that (-)-epicatechin can be used to control long bone elongation in vivo in FGFR3-related diseases.

## Discussion

A growing body of research has identified a number of therapeutic approaches for the treatment of defective bone growth in achondroplasia, the most common form of dwarfism.^2^ Our present results provide strong evidence for a potential therapeutic effect from (-)-epicatechin on the cartilage growth plate and ciliogenesis in FGFR3-related disorders. Over the last few decades, the beneficial effects of polyphenols on human health have been increasingly well documented. Several preclinical studies have demonstrated that (-)-epicatechin is effective against sarcopenia,^36^ alleviates inflammation in lipopolysaccharide-induced lung injury,^37^ prevents the development of dilated cardiomyopathy,^38^ and improves vascular function.^39^ Here, we provide physiological evidence (in cell-based and murine models of ACH) and molecular evidence to show that (-)-epicatechin treatment modifies bone growth. Although the role of fibroblast growth factor (FGF) in skeletal development is quite well understood, little is known about the intracellular signaling that mediates the overactivation of FGFR3. Our data suggest that phosphorylated ERK1/2 and p38 and total Sox9 proteins are regulated in response to FGF signaling. Both ERK1/2 and p38 phosphorylation levels and the Sox9 expression level are elevated in the growth plates of *Fgfr3*^*Y367C/+*^ mice, emphasizing the dysregulation of these proteins in *Fgfr3*-related skeletal dwarfism. Literature data show that the expression of a constitutively active mutant of Mek1 rescues the skeletal overgrowth phenotype in *Fgfr3*-deficient mice and that p38, Sox9 and ERK1/2 upregulate hypertrophic differentiation.^31,40^ Here, we visualized in silico that in the FGFR3 ATP-binding pocket, (-)-epicatechin clearly remained bound to ERK1, ERK2 and p38. Interestingly, in vivo, (-)-epicatechin treatment decreased the activation of p38, ERK1/2, and Sox9 and was associated with a greater hypertrophic chondrocyte volume, which is known to be a major determinant of the longitudinal bone growth rate. We further showed that (-)-epicatechin interacts with the tyrosine kinase pocket of ERK1/2 and p38, which suggests the presence of a direct link to the compound’s effects on the MAPK signaling pathway.

Our results also showed how (-)-epicatechin rescues proliferating columnar chondrocytes with a flattened, stacked appearance. The clonal expansion of chondrocytes resulted in bone growth. At the cellular level, (-)-epicatechin rescued primary cilium elongation defects and thus modified cell division and columnar zone elongation. We therefore examined (-)-epicatechin as a treatment to directly affect growth plate organization and proliferation by modifying the length and the ϕ angle of the primary cilia in the cartilage tissue, thus improving bone growth. We previously demonstrated that sustained FGFR3 activity was associated with shorter cilia,^7,9^ abnormal chondrocyte homeostasis, and disturbance of the growth plate’s columnar organization *via* chondrocyte rotation.^9,10,13^ Our in vitro and in vivo results show that (-)-epicatechin was associated with longer chondrocyte primary cilia. Our morphometric studies of the growth plate showed that (-)-epicatechin treatment modified chondrocyte alignment and the length and position of the primary cilia in the growth plate. These findings were supported by our transcriptomic analysis, highlighting (i) the abnormal expression of several genes involved in ciliogenesis and the Ihh and PKA signaling pathways and (ii) their normalization after (-)-epicatechin treatment. We were therefore not surprised to see that the expression levels of genes linked to skeletal ciliopathies were abnormal in models of ACH compared to controls; these genes included *Dync2li1* (the pathogenic gene in short rib polydactyly syndrome) and *Kif22* (the pathogenic gene in spondyloepimetaphyseal dysplasia with joint laxity).^41,42^

Given the essential role of primary cilia in Hh signaling and the known interactions between members of the FGF and Hh pathways,^3^ it is likely that inhibition of chondrocyte proliferation by upregulated FGF signaling is caused (at least in part) by inactivation of Ihh signaling.^43^ Our transcriptomic data confirmed that the Hh signaling pathway is dysregulated in *Fgfr3*^*Y367C/+*^ mice. Ptch1 is essential for limb development^44,45^ and interaction with Smoothened and Hh signaling through the Gli1 regulator.^46–49^ This upregulation of *Ptch1* is in agreement with data from another mouse model of an *Fgfr3* gain-of-function mutation.^50^ The present study demonstrated that (-)-epicatechin treatment controls the Ihh-related primary cilia signaling pathway by acting on the Ihh/Ptch1/Gli1/Smo cascade in cartilage. We also highlight a putative role of the PKA pathway and *Adcy7* in *Fgfr3*^*Y367C/+*^ growth plate cartilage. Given that PKA was found to regulate Hh signaling in primary cilia and adenylate cyclase was clearly identified in skeletal primary cilium,^51^ we hypothesize that PKA interferes with Ihh signaling in FGFR3-related disorders. Taken together, our results demonstrate that (-)-epicatechin restores the impairments in the various genes controlling cilium elongation by acting on both the Ihh and PKA signaling pathways involved in bone growth regulation.

A better mechanistic understanding of the regulation of primary cilia formation in vivo provides foundations for therapeutic interventions in FGFR3-related disorders. Although the current treatment for ACH restores chondrocyte differentiation *via* the MAPK pathway, it fails to restore the columnar arrangement of chondrocytes.^15,16^ This is in contrast with (-)-epicatechin, which (in addition to chondrocyte differentiation) restores the columnar arrangement by rescuing defects in the primary cilium through regulation of primary cilia-related genes and thus enabling growth plate elongation. These data emphasize the relevance of (-)-epicatechin’s action to control both proliferation and differentiation. We consider that modulation of the FGFR3-activated signaling pathways by (-)-epicatechin provides a rationale for developing treatments for ACH. Moreover, strict control of FGFR3 signaling by (-)-epicatechin provides a rationale for developing alternative therapeutic approaches with (-)-epicatechin alone or in combination with other relevant ACH treatments.

## Materials and methods

### Ethics statement

All animal procedures and protocols were approved by the local animal care and use committee (approval number APAFIS#24826-2018080216094268 v5) and were carried out in compliance with EU directive 2010/63/EU for animals.

### Transcriptomics

Details of the *Fgfr3*^*Y367C/+*^ mouse models with a C57BL/6 background were previously described.^23^ Three pairs of *Fgfr3*^*Y367C/+*^ and *Fgfr3*^*+/+*^ control littermates were produced at four time points (d7, d14, d21, and d28). Mouse genotypes were ascertained by PCR as described previously.^23^ Mouse femoral heads were collected at slaughter and frozen in liquid nitrogen. The samples were crushed, and RNA was extracted using a Qiagen RNeasy Mini kit and Qiagen’s animal tissue protocol. All RNA samples had an RNA integrity number greater than 8 and were analyzed on Affymetrix Mouse Genome 430 2.0 arrays by our service provider (PartnerChip, Evry, France). Processed and raw data were submitted to the Gene Expression Omnibus database (ID: GSE145821). Statistical analyses were performed using BioConductor 2.5, normalized using the gcrma package and were log2-transformed. Probesets differentially expressed by *Fgfr3*^*Y367C/+*^ vs. *Fgfr3*^*+/+*^ animals during their development were identified by computing contrasts with a genotype*timepoint design. To define the gene expression profiles, we also computed the interactions and linear, quadratic and cubic polynomial contrasts. *P* values were adjusted for multiple testing using the eBayes function, and the false discovery rate was computed using the decideTests function. Annotations were retrieved from the MGI database. Gene Ontology annotations were used to identify the genes involved in cilium organization and function. Genes with at least 3 types of proof were also retrieved from Cildb.^24^ Heatmaps were generated online using Morpheus software.

### Chromatography and ESI-TOF mass spectrometry detection

The compounds in the *Theobroma cacao* extract fractions were separated as described previously,^18^ with one modification: the HPLC system was coupled to a TOF mass spectrometer equipped with an ESI interface, operating in negative ion mode with a capillary voltage of +3.5 kV. The optimum values of the other parameters were as follows: drying gas temperature, 200 °C; drying gas flow, 10 L·min^−1^ and nebulizing gas pressure, 2.3 bar. The mass range for detection was 50–1 200 *m/z*. To ensure repeatability, samples were injected in triplicate.

### NMR analyses

Each sample was dissolved in DMSO-d6 and transferred into an oven-dried 5 mm NMR tube. The NMR spectra were recorded at 293 ± 0.1 K on a Bruker Avance III 600 spectrometer operating at a proton frequency of 600.13 MHz and fitted with a 5 mm QCI quadruple resonance pulse field gradient cryoprobe. The multiplicities observed were labeled a s = singlet, d = doublet, dd = doublet of doublets, t = triplet, m = multiplet, and bs = broad singlet. Each sample was measured with 8 dummy scans prior to 128 scans. The acquisition parameters were as follows: size of free induction decay (FID) = 64 K, spectral width = 20.5 ppm, acquisition time = 2.73 s, relaxation delay = 10 s, receiver gain = 20.2, and FID resolution = 0.25 Hz. A presaturation pulse sequence (Bruker 1D noesygppr1d) was used to suppress the residual H_2_O signal *via* irradiation at the H_2_O frequency during the recycling and mixing times. The resulting spectra were automatically phased, baseline-corrected, and calibrated against the trimethylsilyl-2,2,3,3-tetradeuteropropionate signal at 0.0 ppm. The t1 time was set to 4 µs, and the mixing time (d8) was set to 10 ms. The spectrometer transmitter was locked onto the DMSO-d6 frequency. Spectra were processed using TopSpin^TM^ software (version 3.1, Bruker, Germany). 1H–1H total correlation spectroscopy (TOCSY) spectra, 1H-13C heteronuclear single quantum coherence (HSQC) spectra, and 1H-13C heteronuclear multiple bond coherence (HMBC) spectra were recorded using standard Bruker sequences. The TOCSY spectra were obtained by applying a relaxation delay of 2.0 s, a spectral width in both dimensions of 7 194.25 Hz, and a receiver gain of 64.0. These spectra were then processed using a sine-bell window function (shifted sine bell = 2.0). The HSQC spectra were acquired using a relaxation delay of 1.0 s and a spectral width of 7 211.54 Hz in F2 and 24 900.71 Hz in F1. A quadratic sine window function (shifted sine bell = 2.0) was used for HSQC spectra. The HMBC spectra were recorded with the same parameters as those for the HSQC spectra, except that the spectral width in F1 was 37 729.71 Hz. The coupling constants was set to 145 Hz for the HSQC experiments and 145 and 8 Hz (long range) for the HMBC experiments.

### Primary and immortalized human chondrocyte and mouse chondrocyte cultures

Primary and immortalized mutant cells were obtained and cultured as described previously.^9,22^ Human and mouse cells were incubated with FGF2 (100 ng·mL^−1^) for 5 min and then with *Theobroma cacao* extract fraction 5 (100 µg·mL^−1^) or (-)-epicatechin (80 µg·mL^−1^) (Sigma-Aldrich, HW101708-1) for 30 min. Cultured chondrocytes were immunolabeled as previously described.^9^

### Total RNA extraction and RT-qPCR

Reverse transcriptase quantitative polymerase chain reaction (RT-qPCR) was used to evaluate RNA expression after (-)-epicatechin treatment. RNA was extracted using an RNeasy Mini Kit (Qiagen) according to the manufacturer’s instructions. The measured quantity of RNA (500 ng) was reverse-transcribed using SuperScript™ II Reverse Transcriptase (Invitrogen). RT-qPCR was performed with a ViiA-7 system (Applied Biosystems) using SYBR Green (Life Technologies) for fluorescence detection. qPCR optimization was performed to avoid off-target products and artifacts. This optimization was carried out with negative and positive control samples. We also examined the specificity and identity of the amplified product by melting curve and the amplicon size. Primers were designed using the Primer3Plus website. RT-qPCR data were analyzed using the 2^−ΔΔCT^ method, and β-actin was used as the housekeeping control. The primer sequences used are listed in Table 2.

**Table 2:** Primer sequences used

Gene name	Forward 5′ → 3′	Reverse 5′ → 3′
Dync2li1	AGTCAGCAAAGCCGACCTTAGCG	TCCAGCAAGGAGGTTCCTCCACC
Kif24	TCGCCAACATCTCCCCAAGCC	TGGTAGCTGAAGCACAACACTTAACGC
Kif22	AGGGAAGCCAAGGGACCCCC	GTGCCTCCTGGGCCAACACC
Ptch1	CTGGCAGCCGAGACAAGCCC	CCGGTGAGGCCGGATGTTGG
Ihh	GGCGCGCTTAGCAGTGGAGG	CTGGGCTCCGGCAGGAAAGC
Smo	CTGGACCAAGGCCACCCTGC	TTCTGCAGCAGCTCACGCCG
Gli1	CGCGCCAAGCACCAGAATCG	TGTTTGCGGAGCGAGCTGGG
Atp8a2	TGGACCTGGCGCTCTCATGC	CCGATGGCCAGGGTGATGGC
Prkar2b	ACCTCTCCTGGTGCTCTGTGGG	CTTGAGGAACGGCAGGGACTCG
Adcy7	GGCCTTGGTGGCCTACCTGC	CGGAGCAAGGGTGTGGCTGG
β-Actin	CATGTTTGAGACCTTCAACAC	GCCATCTCCTGCTCGAAGTCTAG

### Transient transfections with the cDNA FGFR3 constructs

HEK293 cells at 70%–80% confluence were transiently transfected with FGFR3 human constructs (FGFR3-Y373C, FGFR3-G380R, FGFR3-K650E). Transfected cells were incubated with (-)-epicatechin overnight and then lysed in RIPA buffer (50 mmol·L^−1^ Tris-HCl pH 7.6, 150 mmol·L^−1^ NaCl, 0.5% NP40, and 0.25% sodium deoxycholate, supplemented with protease and phosphatase inhibitors; Roche). Proteins were immunoprecipitated by the addition of 3 μL of rabbit anti-FGFR3 (Sigma-Aldrich, F0425) per 500 μg of protein with protein A-agarose (Roche). The excised FGFR3 protein was eluted and then denatured at 95 °C for 10 min in NuPAGE LDS sample buffer (Life Technologies, NP0008) and 2.5% β-mercaptoethanol.

### Immunoblot analysis

Whole-cell lysates and immunoprecipitated proteins were subjected to NuPAGE 4%–12% bis-tris acrylamide gel electrophoresis (Life Technologies). Using standard protocols, we probed the blots with the following primary antibodies: anti-phosphotyrosine (Cell Signaling Technology), anti-phosphorylated ERK1/2 (Thr180/Tyr182) (Cell Signaling Technology, #4370), anti-total ERK1/2 (Sigma-Aldrich, M5670), anti-phosphorylated p38 (Thr180/Tyr182) (Cell Signaling Technology, #9205), anti-total p38 (Cell Signaling Technology, #2708), anti-total PCNA (Abcam, ab29-100), and anti-β-actin (Millipore, MAB1501). Proteins were immunodetected with an anti-rabbit IRDye^®^ 800CW antibody (LI-COR Biosciences, 926-32211) and an anti-mouse IRDye^®^ 680RD antibody (LI-COR Biosciences, 926-68070). Immunoblots were quantified with ImageStudioLite software (LI-COR Biosciences).

### Computational details

The starting coordinates of ERK1, ERK2, and p38 were downloaded from the Protein Data Bank with the following respective accession codes: 4QTB, 5NGU, and 4L8M. The ligand (-)-epicatechin was built using Maestro software and optimized with Amber software. Molecular docking calculations were performed with AutoDock 4.2 software. We selected the docking pose that presented the lowest free energy of binding in the most populated cluster. The stability of the complexes formed by (-)-epicatechin and the kinases ERK1, ERK2, p38, and FGFR3 were further explored through molecular dynamics simulations using Amber software and the FF14SB/gaff2 force field. For each system, the simulation started with energy optimization of the water solvent followed by an overall minimization and a heating phase (from 0 to 300 K). Next, molecular dynamics was performed with the NTP ensemble, leading to a 50 ns trajectory. After the conventional 50 ns period of molecular dynamics, all systems underwent an additional 50 ns of Gaussian accelerated molecular dynamics.

### Ex vivo femur culture system

Femurs were cultured ex vivo as described previously.^22^ The left femur was cultured in the presence of *Theobroma cacao* extract fraction 5 (10 µg·mL^−1^), procyanidin C1 (10 µg·mL^−1^) or (-)-epicatechin (10 µg·mL^−1^) and compared with the untreated right femur. The bone length was measured on Day 0 (D0) and Day 6 (D6).

### Histological and immunohistochemical analyses of paraffin sections

Femur explants were fixed in 4% paraformaldehyde and embedded in paraffin. Serial 5 μm sections were stained with hematoxylin–eosin-safran reagent using standard protocols. For immunohistochemical assessment, sections were labeled with the following antibodies and a Dako Envision Kit: anti-Col X (BIOCYC, N.2031501005; 1:50 dilution), anti-Sox9 (polyclonal antibody, Santa Cruz Biotechnology Inc., catalog D0609; dilution 1:75), anti-phosphorylated ERK1/2 (Thr180/Tyr182) (Cell Signaling Technology, #4370; 1:100 dilution), anti-phosphorylated p38 (Abcam, Ab4822; 1:200 dilution), and anti-Ki-67 (Abcam, Ab16667; 1:3 000 dilution). Images were captured with an Olympus PD70-IX2-UCB microscope and quantified using cellSens software.

### Histological and immunohistochemical analyses of frozen sections

Femur explants or femurs isolated from the mice at P16 were fixed in methanol chilled at −20 °C for 5 h or 24 h, respectively. After incubation in 0.5 mol·L^−1^ EDTA, pH 8 for 72 h or 2 weeks, femurs were placed in 30% sucrose for 24 h, transferred to OCT compound at room temperature, and frozen in isopentane at −45 °C. The 50 μm tissue sections were permeabilized with 0.3% Triton X-100 for 30 min and immunolabeled with rabbit IgG anti-Arl13b (Proteintech #17711-1-AP, IF 1:100) or mouse IgG_1_ anti-γ-tubulin (Sigma-Aldrich #T6557, 1:100) primary antibodies. The primary antibodies were detected with goat anti-mouse IgG_1_ coupled to Alexa Fluor 488 (Life Technologies, 1:400) and anti-rabbit IgG coupled to Alexa Fluor 647 (Life Technologies, 1:400). Tissue sections were mounted with DAPI-Fluoromount G^®^ (CliniSciences). Three-dimensional images of the growth plate were obtained using a spinning disc confocal microscope. Images were displayed using FIJI and the FigureJ plugin.

### Mouse model and drug treatments

The construction of the *Fgfr3*^*Y367C/+*^ mouse model using mice with a C57BL/6 background has been described previously.^22^ Cartilage and bone analyses were performed on 16-day-old mice. The *Fgfr3*^*Y367C/+*^ mice were 1-day old upon treatment initiation and received daily subcutaneous administrations of (-)-epicatechin (Sigma-Aldrich, HW101708-1) (0.1 mg·kg^−1^ body weight) or vehicle (3.5 mmol·L^−1^ HCl, 0.1% DMSO) for 2 weeks. Long bones were measured using a caliper (VWRi819-0013, VWR International).

### Statistical analysis

Differences between experimental groups were assessed by analysis of variance with Tukey’s post hoc test or a Mann–Whitney *U* test. The threshold for statistical significance was set to *P* ≤ 0.05. Statistical analyses were performed using GraphPad Prism software. A paired Student’s *t* test was used to compare two treatments in the same cell population. An unpaired Student’s *t* test was used to compare groups of mice or different primary chondrocyte preparations.

## Supplementary information

Supplementary Figure 1

Supplementary Figure 2

Supplementary Figure 3

Supplementary Figure 4

Supplementary Figure 5

Supplementary Figure 6

Supplementary Figure 7

Supplementary Figure 8

Supplementary Figure 9

Supplementary Table 1

Supplementary Table 2

Molecular simulation of (-)-epicatechin from FGFR3 kinase
