# Supplementary figures and images for: Theobroma cacao improves bone growth by modulating defective ciliogenesis in a mouse model of achondroplasia
Source: Bone Res. 2022 Jan 25;10:8. doi: 10.1038/s41413-021-00177-7 (PMC8789790; doi:10.1038/s41413-021-00177-7)

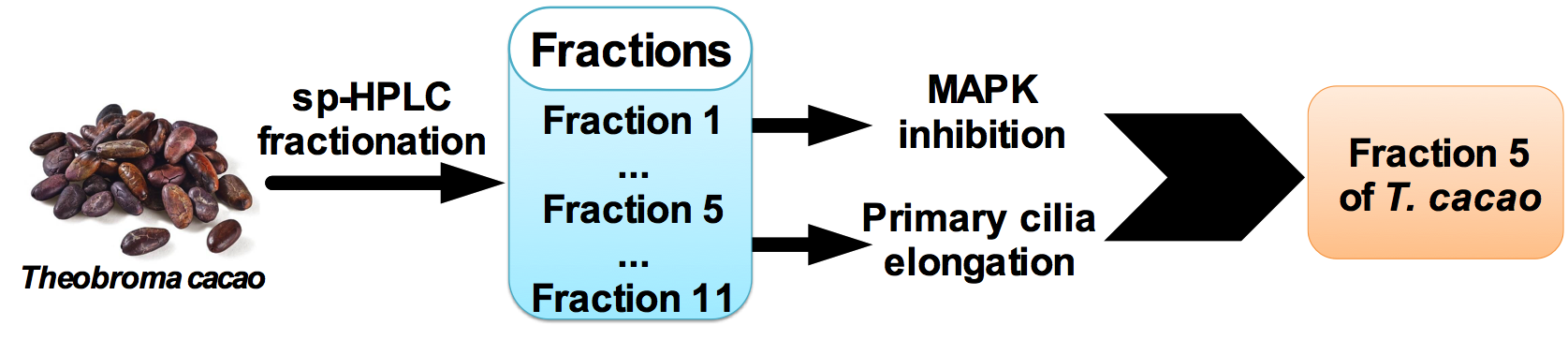

Supplement: Supplementary file 1 — Supplementary Figure 1 [file 41413_2021_177_MOESM1_ESM.tif]

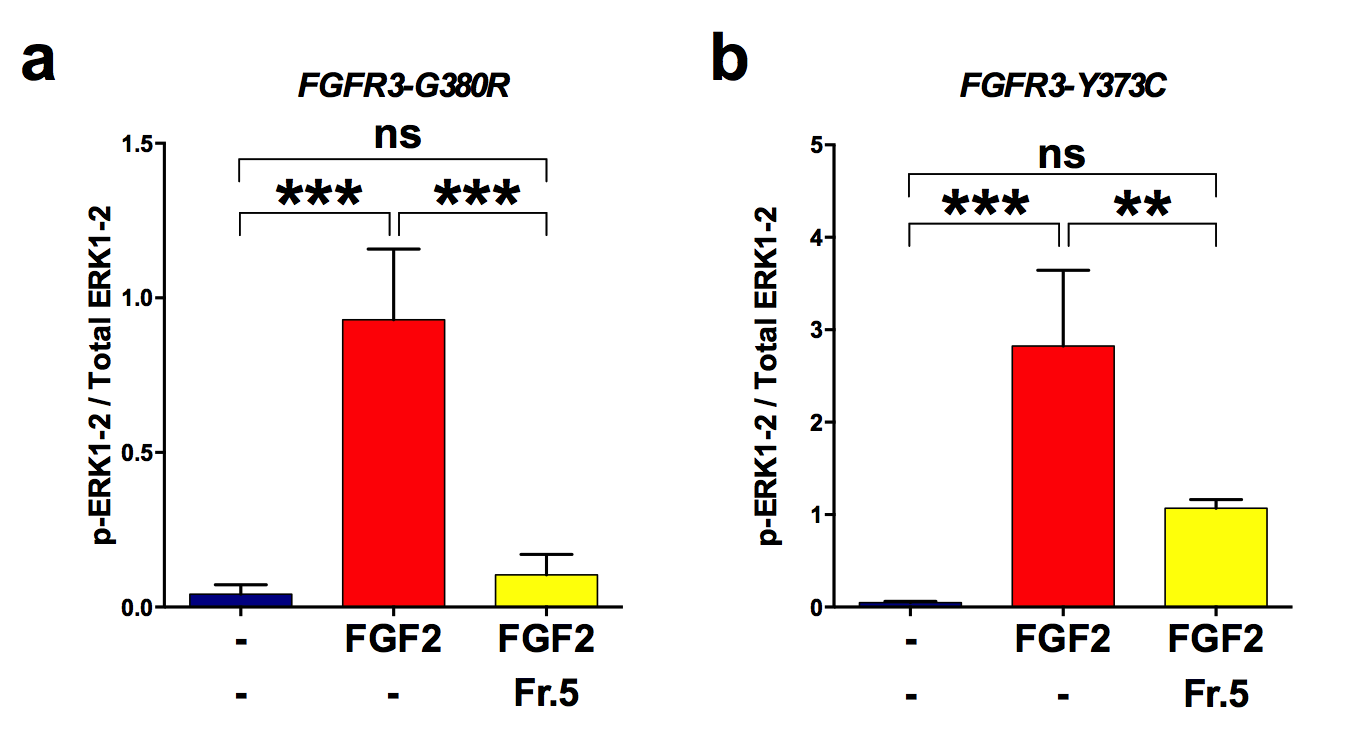

Supplement: Supplementary file 2 — Supplementary Figure 2 [file 41413_2021_177_MOESM2_ESM.tif]

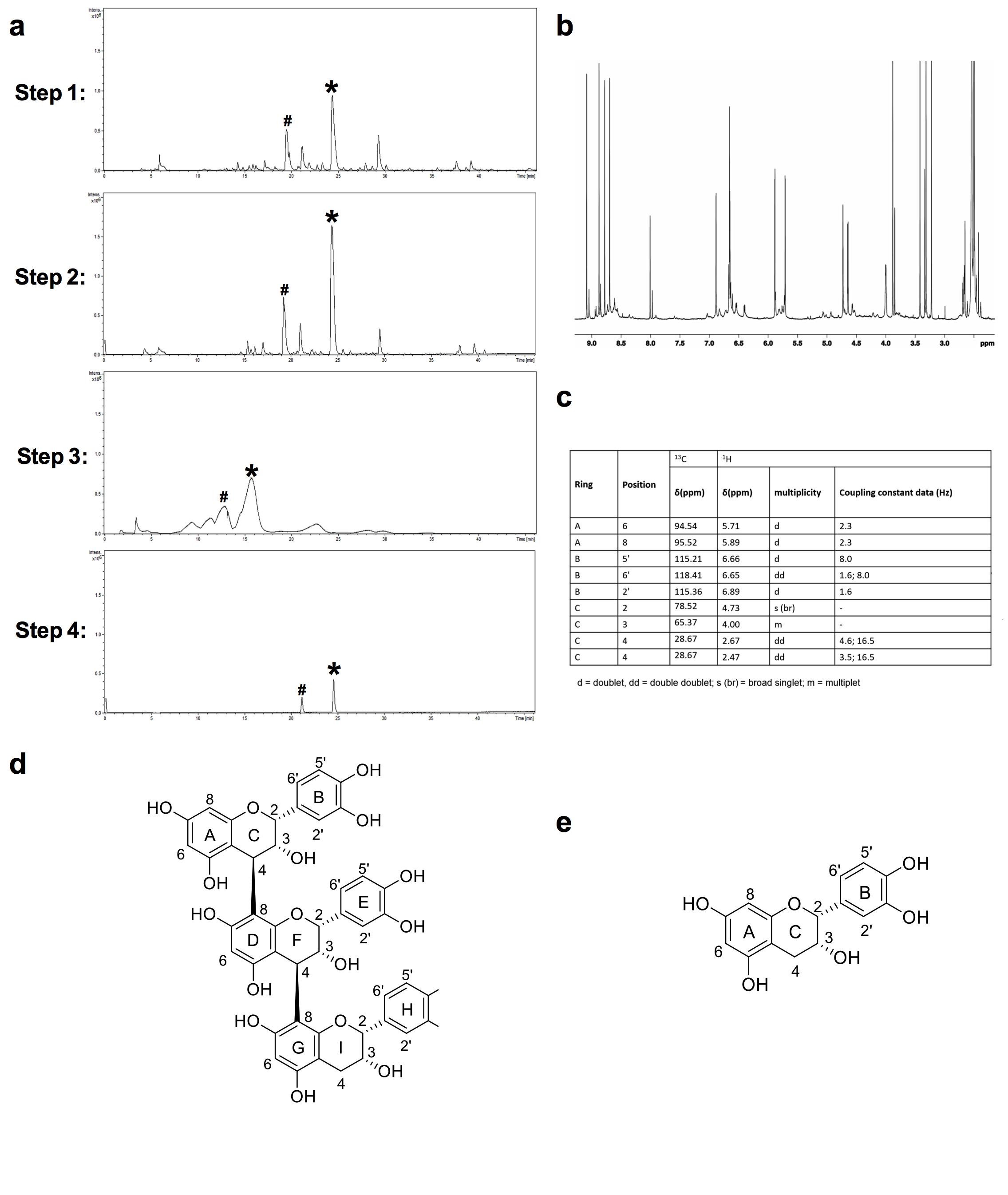

Supplement: Supplementary file 3 — Supplementary Figure 3 [file 41413_2021_177_MOESM3_ESM.tif]

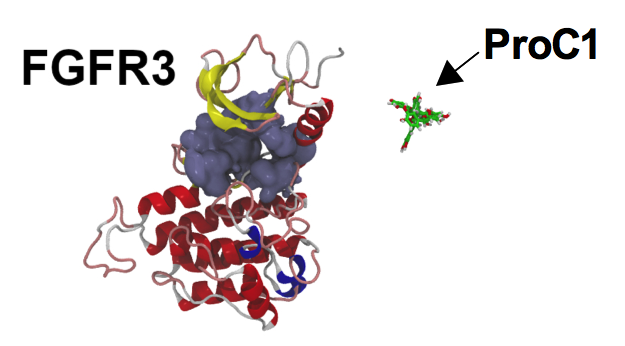

Supplement: Supplementary file 4 — Supplementary Figure 4 [file 41413_2021_177_MOESM4_ESM.tif]

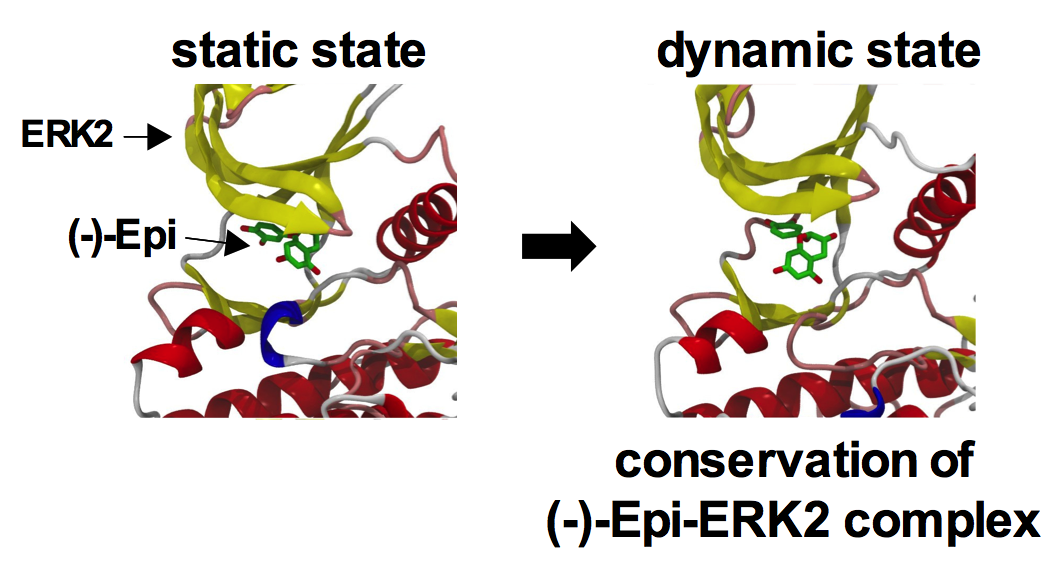

Supplement: Supplementary file 5 — Supplementary Figure 5 [file 41413_2021_177_MOESM5_ESM.tif]

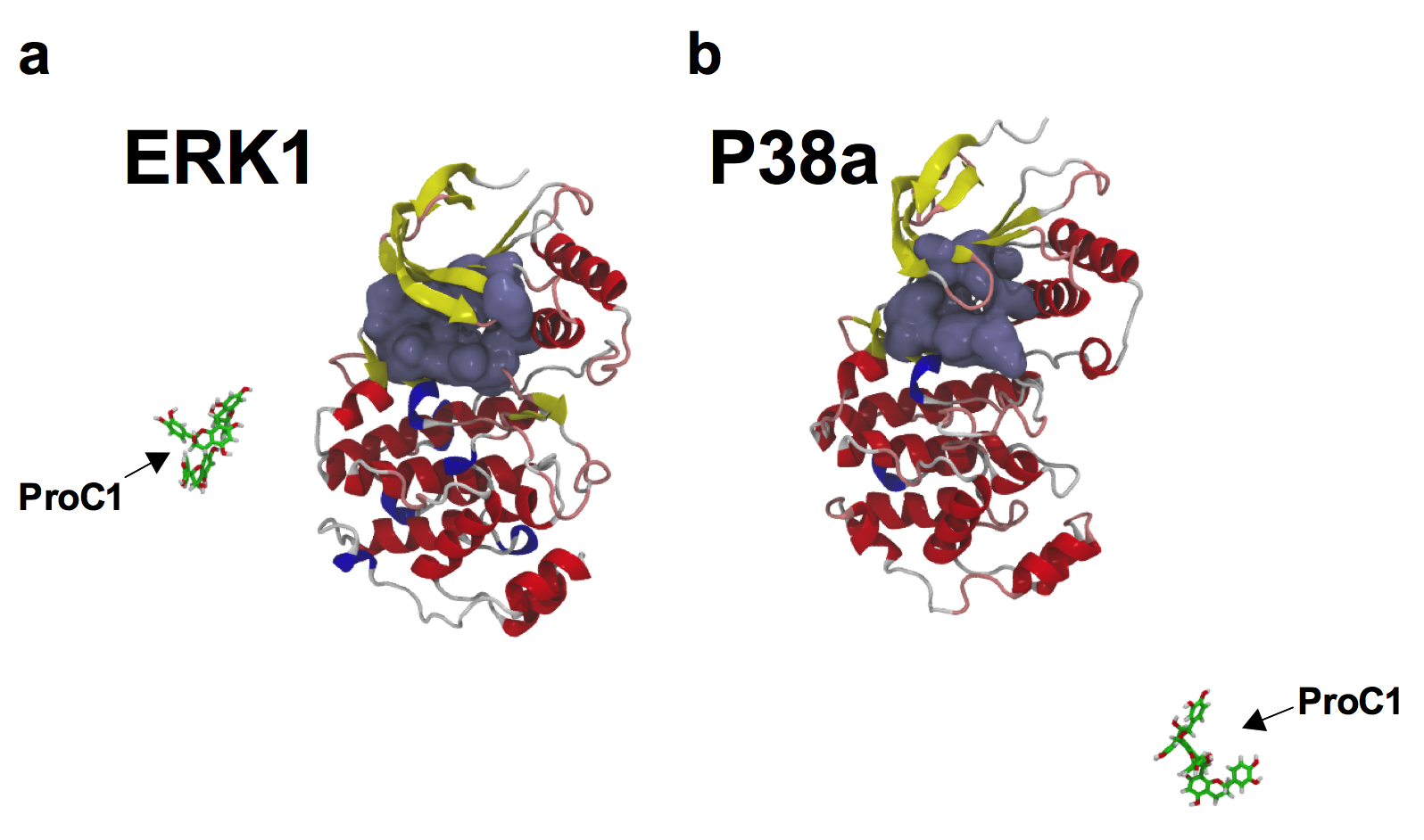

Supplement: Supplementary file 6 — Supplementary Figure 6 [file 41413_2021_177_MOESM6_ESM.tif]

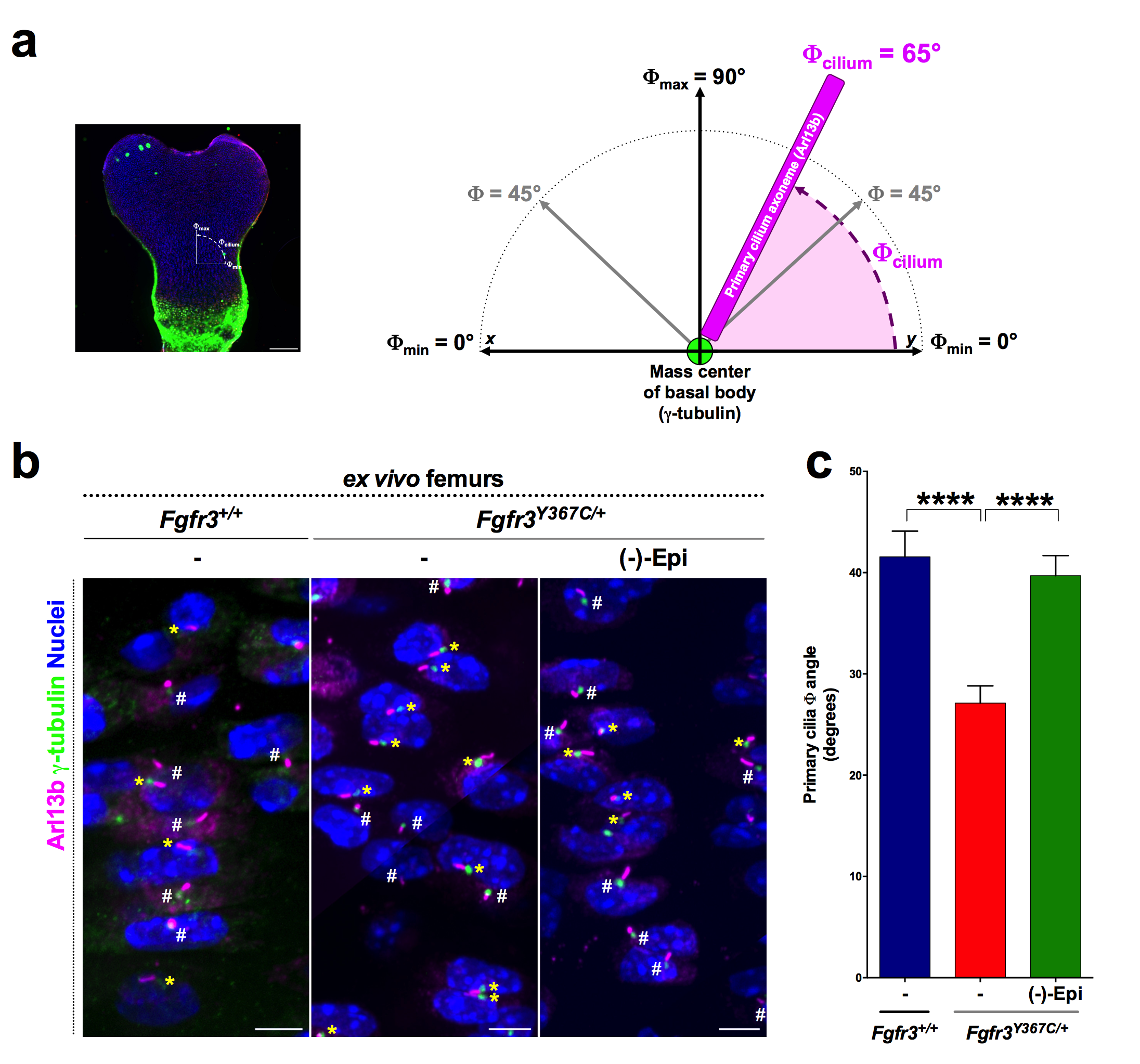

Supplement: Supplementary file 7 — Supplementary Figure 7 [file 41413_2021_177_MOESM7_ESM.tif]

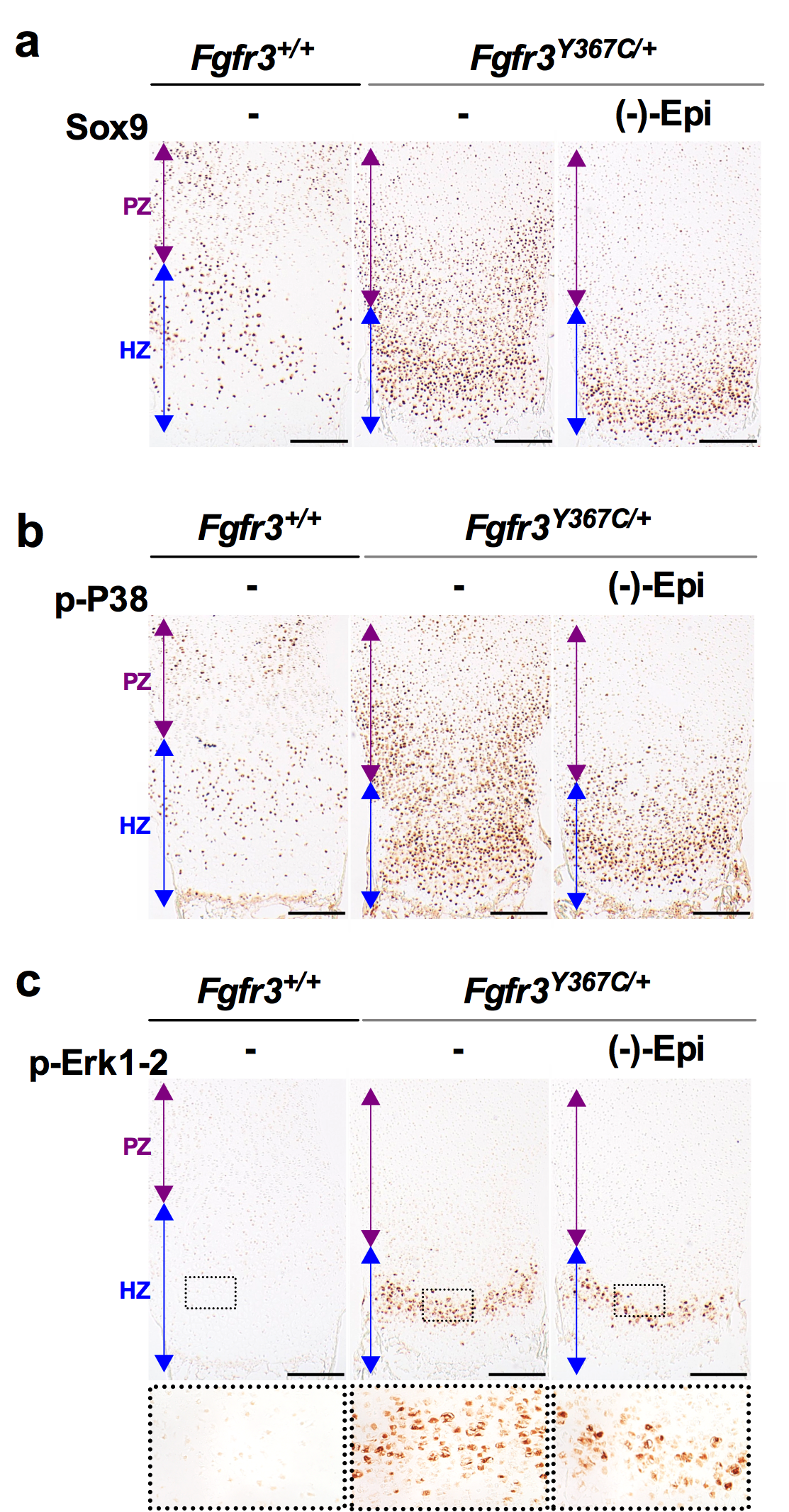

Supplement: Supplementary file 8 — Supplementary Figure 8 [file 41413_2021_177_MOESM8_ESM.tif]

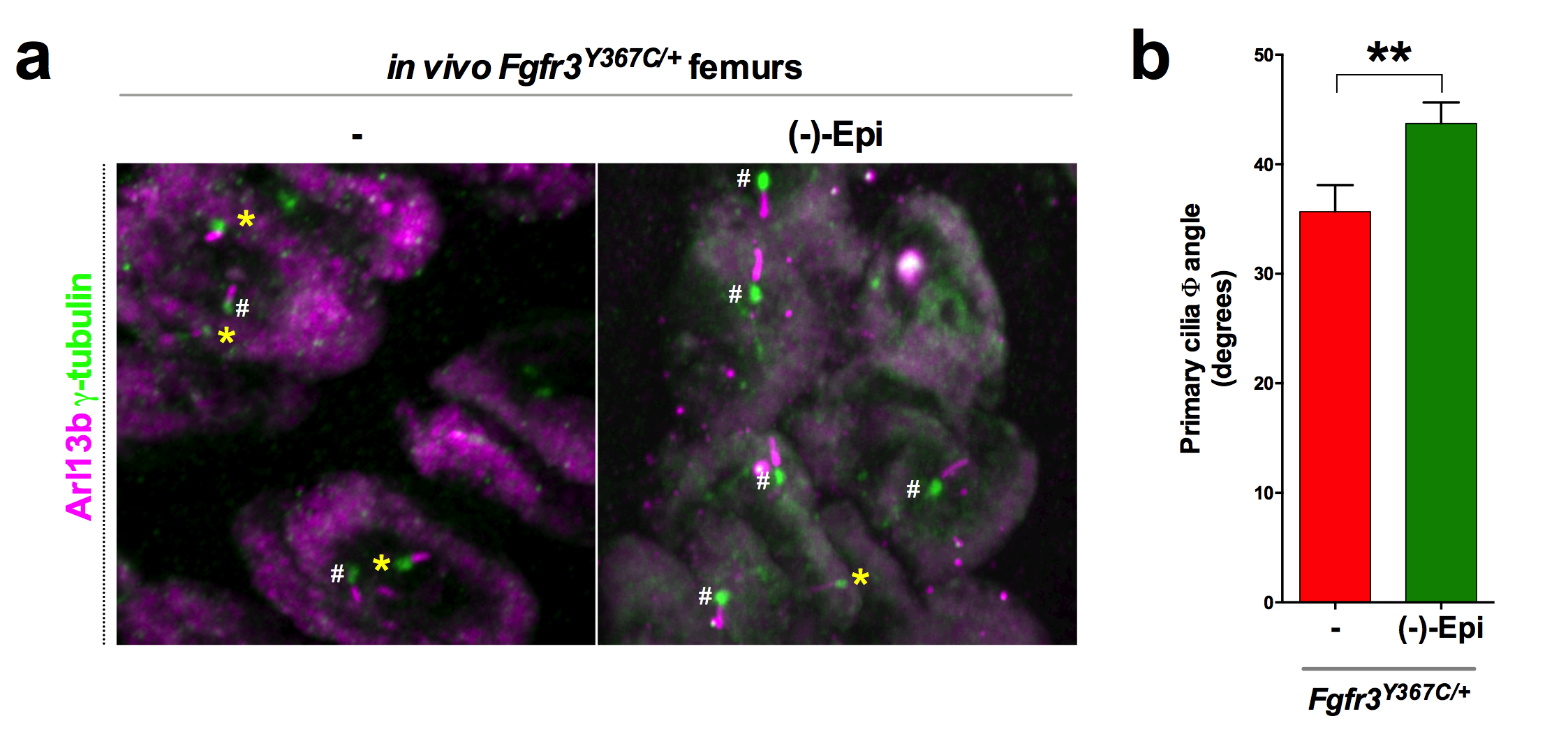

Supplement: Supplementary file 9 — Supplementary Figure 9 [file 41413_2021_177_MOESM9_ESM.tif]
