# Supplementary material for: Theobroma cacao improves bone growth by modulating defective ciliogenesis in a mouse model of achondroplasia
Source: Bone Res. 2022 Jan 25;10:8. doi: 10.1038/s41413-021-00177-7 (PMC8789790; doi:10.1038/s41413-021-00177-7)
Supplement: Supplementary file 11 — Supplementary Table 2 [file 41413_2021_177_MOESM11_ESM.docx]

**Supplementary Table 2. Docking binding energies (in kcal.mol^-1^) along with the percentage of cluster for procyanidin C1 and the FGFR3, ERK1, ERK2 and P38 kinases.**
